# Genome-Wide Identification and Characterization of Pectin Methylesterase Inhibitor Genes in *Brassica oleracea*

**DOI:** 10.3390/ijms19113338

**Published:** 2018-10-26

**Authors:** Tingting Liu, Hui Yu, Xingpeng Xiong, Youjian Yu, Xiaoyan Yue, Jinlong Liu, Jiashu Cao

**Affiliations:** 1 Laboratory of Cell and Molecular Biology, Institute of Vegetable Science, Zhejiang University, Hangzhou 310058, China; 11416009@zju.edu.cn (T.L.); 21616082@zju.edu.cn (H.Y.); 11216053@zju.edu.cn (X.X.); 11516008@zju.edu.cn (X.Y.); 2 Key Laboratory of Horticultural Plant Growth, Development and Quality Improvement, Ministry of Agriculture, Hangzhou 310058, China; 3 Zhejiang Provincial Key Laboratory of Horticultural Plant Integrative Biology, Hangzhou 310058, China; 4 Department of Horticulture, College of Agriculture and Food Science, Zhejiang A & F University, Lin’an 311300, China; yjyu@zafu.edu.cn; 5 Laboratory of Molecular Biology and Gene Engineering, School of Life Sciences, Nanchang University, Nanchang 330031, China; ljl014@sina.com

**Keywords:** *Brassica oleracea*, BoPMEI genes, whole-genome triplication, evolution, expression profiles

## Abstract

The activities of pectin methylesterases (PMEs) are regulated by pectin methylesterase inhibitors (PMEIs), which consequently control the pectin methylesterification status. However, the role of PMEI genes in *Brassica oleracea*, an economically important vegetable crop, is poorly understood. In this study, 95 *B. oleracea* PMEI (BoPMEI) genes were identified. A total of 77 syntenic ortholog pairs and 10 tandemly duplicated clusters were detected, suggesting that the expansion of BoPMEI genes was mainly attributed to whole-genome triplication (WGT) and tandem duplication (TD). During diploidization after WGT, BoPMEI genes were preferentially retained in accordance with the gene balance hypothesis. Most homologous gene pairs experienced purifying selection with ω (*Ka*/*Ks*) ratios lower than 1 in evolution. Five stamen-specific BoPMEI genes were identified by expression pattern analysis. By combining the analyses of expression and evolution, we speculated that nonfunctionalization, subfunctionalization, neofunctionalization, and functional conservation can occur in the long evolutionary process. This work provides insights into the characterization of PMEI genes in *B. oleracea* and contributes to the further functional studies of BoPMEI genes.

## 1. Introduction

The structure of plant cell wall is complicated and includes polysaccharides and proteins with important physical and chemical properties related to the growth and development of plants [1]. Pectins are the main components of polysaccharides in plant cell wall and can be divided into homogalacturonan, xylogalacturonan, rhamnogalacturonan I, and rhamnogalacturonan II according to their different structures [2]. Homogalacturonan is composed of α-1,4-linked galacturonic acid residues and is the most abundant pectin polymer. Homogalacturonan is synthesized in the Golgi complex, secreted to the cell wall as massive methyl-esterified polymers [3], and is demethylesterified by a large enzyme family, namely, pectin methylesterases (PMEs) [4]. The activity of PMEs can result either in cell wall loosening or stiffening, thereby affecting the shape and growth of plants [5]. PME activity is specifically modulated by its proteinaceous inhibitor, namely, PME inhibitors (PMEIs), which can bind to the active site of PMEs to form a stoichiometric non-covalent 1:1 complex [6,7].

PMEIs were first discovered in ripe kiwi fruit [8] and later in many other plants, such as *Arabidopsis thaliana* [9,10,11], broccoli [12], flax [13], maize [14], rice [15], tomato [16], and wheat [17]. Similar to PMEs, PMEI family was also large with up to 49, 79, and 95 members discovered in rice [18], *Arabidopsis* [19], and flax [13], respectively. However, the physiological function of PMEI genes is still largely unknown. In *Arabidopsis thaliana*, only 12 PMEI genes have been identified with a variety of functions. For example, *AtPMEI1* and *AtPMEI2*, the first two identified *A. thaliana* PMEI (AtPMEI) genes, are related to pollen tube growth [9]. Overexpression of an AtPMEI gene, namely, *AtPMEI5*, changes the sensitivity of seed germination to ABA and thus affects the speed of seed germination [20]. AtPMEI10, AtPMEI11, and AtPMEI12 are functional PMEIs that can be induced in *Arabidopsis* during *B. cinerea* infection [11]. In Chinese cabbage, *Bra019903*, *Bra014099*, and *Bra032239*, three stamen-specific genes, might play important roles in male sterility [19]. Antisense expression of *Bol031283*, a *broccoli* PMEI gene, can retard pollen tube growth, resulting in the partial male sterility of the transgenic *Arabidopsis* plants [12]. The PMEI gene found in *Solanum lycopersicum* (*SolyPMEI*) exerts its effect during fruit development [16].

*Brassica oleracea*, an economically important vegetable worldwide, shares a common ancestor with *Arabidopsis* [21,22]. Approximately 13–17 million years ago (MYA), *B. oleracea* experienced a whole-genome triplication (WGT) event, leading to the divergence between its genome and that of *Arabidopsis* [23]. After the WGT event and diploidization, substantial gene fractionation occurred in the genome of *B. oleracea* [24]; during the long period of evolution, the duplicated genes underwent different fates, including pseudogenization, subfunctionalization, neofunctionalization, and functional conservation [25]. With the typical WGT event, its close relationship with *A. thaliana*, and the accomplishment of genome sequencing, *B. oleracea* became an exemplary model for investigating the molecular evolution of gene families [24]. Although the PMEI gene family has been discovered in many species [13,18,26], its existence in *B. oleracea* remains poorly understood.

In this work, we presented a genome-wide identification of the *B. oleracea* PMEI (BoPMEI) genes. Detailed analyses of the BoPMEI gene family, including chromosomal locations, conserved motifs, phylogenetic relationships, gene structures, physicochemical properties, and *cis*-elements, were performed. In addition, the synteny and retention rates were evaluated to reflect the Influences of WGT on the expansion of BoPMEI gene family. The nonsynonymous substitution rate (*Ka*), synonymous substitution rate (*Ks*), and ω (*Ka*/*Ks*) ratio were calculated to determine the divergence time and selection pressure of homologous gene pairs. Furthermore, the expression patterns of BoPMEI genes were characterized in different tissues. The systematical analysis of PMEI gene family in *B. oleracea* can serve as a basis for further studies on plant PMEI genes.

## 2. Results

### 2.1. Identification of PMEI Genes in the Genome of B. oleracea

First, BLASTP searches were conducted in BRAD (Brassica database) to investigate the putative BoPMEI members by using 79 AtPMEI protein sequences [19] as queries downloaded from TAIR (The Arabidopsis Information Resource). A total of 158 non-redundant sequence hits in BRAD were identified. In addition, 169 putative BoPMEI protein sequences were found with HMMER 3.0 software by using the global hidden Markov model (HMM) profile of the conserved PMEI domain (Pfam04043). After removing the redundant hits obtained from these two methods, the remaining sequences were investigated by SMART and Pfam to filter the genes that did not cover the conserved domain of PMEI gene family. Finally, 95 PMEI members were identified in the genome of *B. oleracea* (Table S1).

### 2.2. Genomic Distribution and Tandem Array

**Figure 1:**
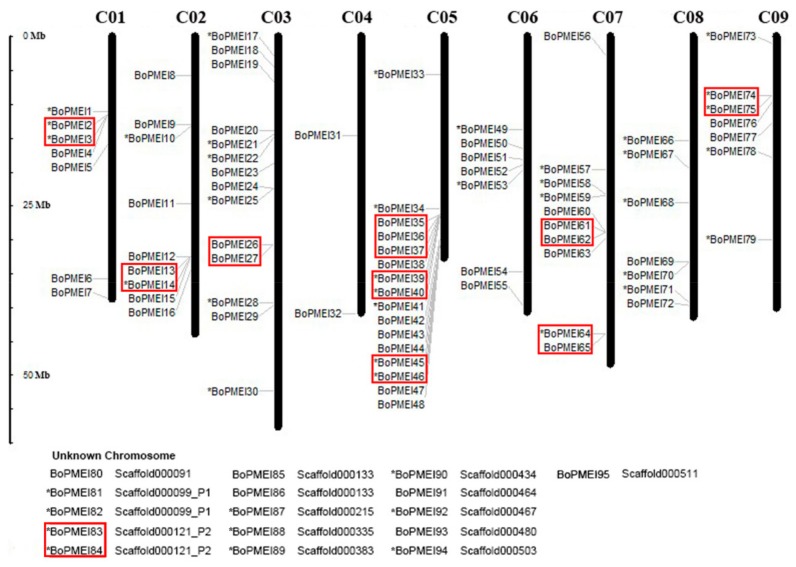
Chromosomal locations of PMEI genes in *Brassica oleracea*. The chromosome numbers are represented at the top of each chromosome. The asterisks next to gene names indicate the sense strands, and the gene names without asterisks indicate that these genes localize at the 3′ to 5′ direction. Tandem arrays of BoPMEI are marked with the red rectangle. Sixteen BoPMEI genes could not be mapped onto a specific chromosome.

The analysis of chromosomal locations showed that *BoPMEI1*–*BoPMEI79* was apparently unevenly distributed on nine chromosomes, and the remaining 16 unanchored genes were mapped on scaffolds (Figure 1). The number of BoPMEI genes per chromosome possessed was from 2 to 16, with chromosome 4 harboring only 2 and chromosome 5 having up to 16. In addition, 44 BoPMEI genes were in the 5′ to 3′ direction, and the rest were localized in the opposite direction.

Among all the BoPMEI genes identified in the genome of *B. oleracea*, 10 tandemly duplicated clusters containing 21 BoPMEI genes were found and were irregularly mapped on chromosomes 1, 2, 3, 5, 7, 9, and Scaffold000121_P2; among which, chromosomes 5 and 7 had three and two clusters, respectively (Figure 1). Only one cluster covered three BoPMEI genes, whereas the remaining nine clusters covered two. However, in *A. thaliana,* 28 out of 79 PMEI genes were tandemly duplicated (Figure S1). In particular, the chromosome 5 of *A. thaliana* contained a cluster harboring up to seven PMEI members (*At5G46930*-*At5G46940*-*At5G46950*-*At5G46960*-*At5G46970-At5G46980*-*At5G46990*).

### 2.3. Phylogenetic and Conserved Motif Analysis

**Figure 2:**
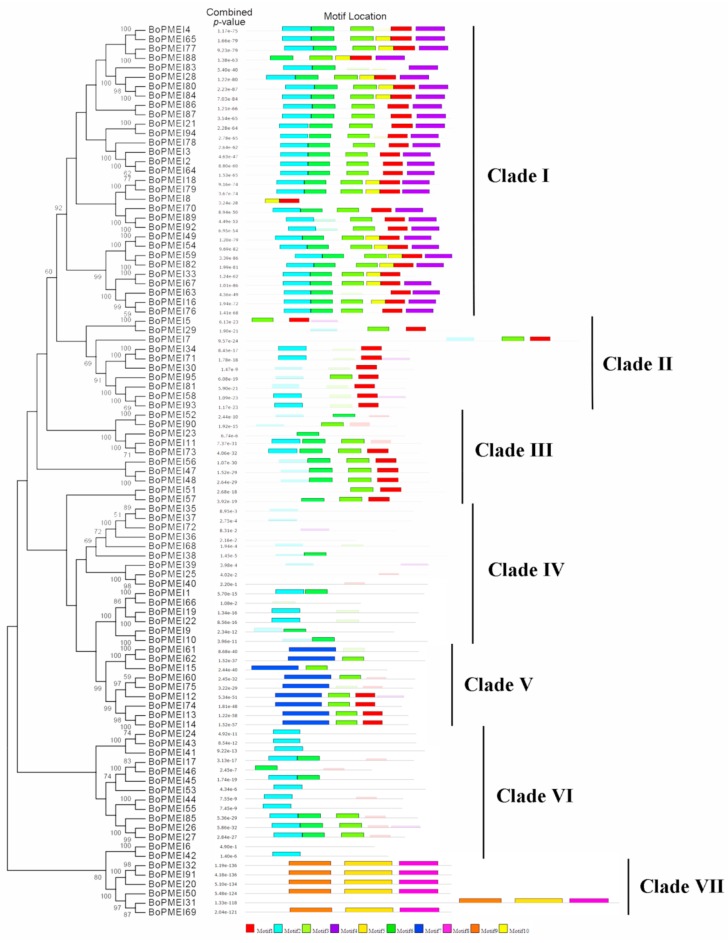
Phylogenetic and conserved motif analyses of PMEI proteins in *Brassica oleracea*. Phylogenetic tree of PMEI proteins was constructed by neighbor-joining with 1000 bootstraps in MEGA 6.0. Bootstrap values lower than 50 are not displayed. The MEME software was used to analyze the conserved motifs as described in the methods. The vertical dark bars on the right part represent seven clades.

A phylogenetic tree was constructed to investigate the evolutionary relationships among the PMEI genes in *Brassica oleracea* (Figure 2). Ten conserved motifs were identified by the MEME program to detect the motifs shared among the PMEI protein sequences in *B. oleracea* (Figure 2 and Figure S2). In the phylogenetic tree, the BoPMEI family members were classified into seven clades with 31, 10, 10, 15, 9, 14, and 6 members, respectively. Most of the members of a given group had a similar motif composition (Figure 2). The widths of the motifs were in the range of 6–50 amino acids, and each of the BoPMEI protein sequences contained 0–6 conserved motifs. The number of sites contributing to the construction of motifs varied from 6 (motifs 5, 8, and 10) to 50 (motifs 1, 2, and 3). Most of the BoPMEI paralogs, such as BoPMEI20/BoPMEI32/BoPMEI91, shared similar composition and distribution of motifs, thus suggesting that function was conservation among them. By contrast, a small number of paralogous genes, such as BoPMEI6/BoPMEI42, had different motif compositions, thus implying functional differentiation among these BoPMEI genes during evolution. In addition, two BoPMEI genes had no conserved motif, and eight BoPMEI genes contained only one or two scanned sites (sites detected using a motif scanning algorithm). This finding indicates that these BoPMEI genes might experience function loss during the evolution of BoPMEI gene family.

### 2.4. Characterization of Gene Structure and Physicochemical Property

To determine the structures of exon and intron of each BoPMEI gene, we compared the coding sequences with their corresponding genomic DNA sequences. Given that most of the BoPMEI genes had no introns in their DNA sequences, we only observed 16 introns with 62.5%, 25.0%, and 12.5% in phases 0, 1, and 2, respectively (Figure S3). A total of 111 exons were detected in the BoPMEI genes. The average exon length was 478.73 bp, and each gene contained 1.17 exons on average (Table S1). These data were similar to those for AtPMEI genes. In *Arabidopsis*, 24 introns were identified in PMEI genes with 70.8%, 16.7%, and 12.5% in phases 0, 1, and 2, respectively (Figure S4). A total of 103 exons were discovered in AtPMEI genes. The average exon length was 444.84 bp, and each gene contained 1.30 exons on average (Table S2). In addition, the number of peptides encoded by the 95 BoPMEI genes ranged from 80 aa (BoPMEI8) to 393 aa (BoPMEI31), and the estimated molecular weights were from 9.01 kDa to 43.04 kDa. The theoretical isoelectric points varied from 3.93 (BoPMEI7) to 11.24 (BoPMEI30). Seventy-nine BoPMEI proteins covered a signal peptide sequence, which were 16–31 amino acids in length. Twenty-two BoPMEI proteins had a 100% percent chance of solubility when overexpressed in *Escherichia coli* (Table S1). Thirty-one BoPMEI proteins had a transmembrane helix. Plant-mPLoc predicted 91 BoPMEIs to be secreted to the cell membrane. Using the software WOLF PSORT, 81 BoPMEIs were predicted to be targeted either extracellularly or to the plasma membrane, whereas ProtComp 9.0 predicted 95 BoPMEIs are extracellular (Table S1). In Arabidopsis, the PMEI proteins were also predicted to be secreted to be extracellular (Table S2). The predicted locations of BoPMEIs and AtPMEIs were similar to the results in previous studies; moreover, the results will be verified through experiments in further study [13,18].

### 2.5. Analysis of Synteny and Retained Proportion

**Figure 3:**
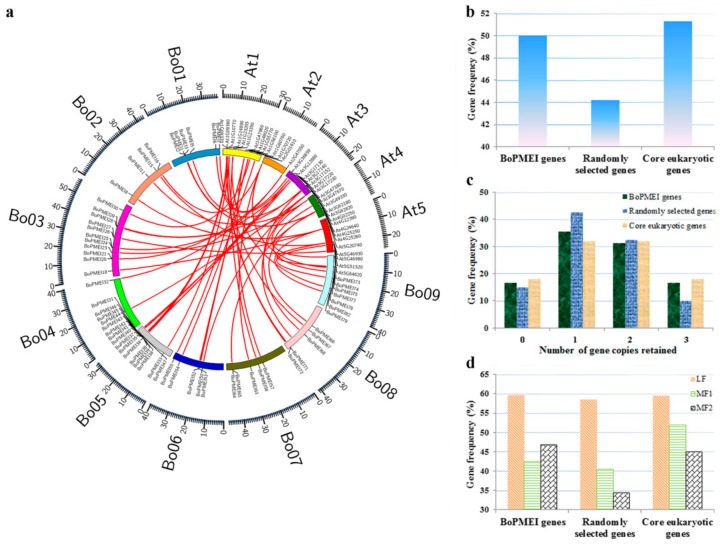
Synteny and retention of BoPMEI genes. (**a**) Syntenic analysis of PMEI genes in *Brassica oleracea* and *Arabidopsis thaliana*. Nine *B. oleracea* chromosomes and five *A. thaliana* chromosomes are colored with different random colors. Sixteen syntenic PMEI ortholog pairs are not shown, because these BoPMEI genes could not be mapped onto a specific chromosome of *B. oleracea*. (**b**) Retained rates of BoPMEI genes, randomly selected genes, and core eukaryotic genes. (**c**) Retention by number of homologous copies in the syntenic region. (**d**) Retained rates of BoPMEI genes, randomly selected genes, and core eukaryotic genes among the three *B. oleracea* subgenomes.

In our study, more PMEI genes were detected in *B. oleracea* than in *A. thaliana*, which shared a common ancestor with the former. The syntenic ortholog pairs between BoPMEI and AtPMEI genes were recognized by searching “syntenic gene” in BRAD to explore the potential duplication mechanism underlying the expansion of BoPMEI genes [27]. The result showed that up to 77 pairs of orthologous PMEI genes were found in collinear genomic blocks that reformed after WGT (Figure 3a and Table S3), indicating that the expansion of PMEI genes in *B. oleracea* mainly relied on the WGT event.

In addition, we discovered three nonsyntenic PMEI ortholog pairs by searching “nonsyntenic orthologs” in BRAD [27] and one gene pair that showed high identities (>70%) (Table S3). The orthologous genes of 20 AtPMEI loci cannot be identified in *B. oleracea*. Further analyses on the orthologs of AtPMEI loci in other Brassicaceae species were performed to determine whether these loci were lost in *B. oleracea* or were newly generated in *A. thaliana*. Brassicaceae species were divided into three lineages according to previous studies [28,29]. Lineage I included *A. thaliana*, *A. lyrata*, *Capsella rubella*, *C. sativa* and *Leavenworthia alabamica*. Lineage II covered *B. rapa*, *B. napus*, *B. oleracea*, *Thellungiella halophile*, *T. salsuginea*, *Sisymbrium irio*, and *Schrenkiella parvula*. The analysis results indicated that eight loci of AtPMEI (*At1G11362*, *At1G11593*, *At1G56620*, *At2G31425*-*At2G31430*-*At2G31432*, *At3G05741*, *At4G15750*, *At5G38610*, and *At5G50030*-*At5G50040*-*At5G50050*-*At5G50060*-*At5G50070*) existed in the species from both Lineages I and II (Table S4). This finding suggests that these AtPMEI loci might disappear in *B. oleracea*. In particular, *At2G31425*-*At2G31430*-*At2G31432* and *At5G38610* were also found in the primitive Brassicaceae species, namely, *Aethionema arabicum*. Thus, we speculated that these loci appeared in the Brassicaceae species that emerged before *A. arabicum* but were lost in the evolution of *B. oleracea*. Among the remaining AtPMEI loci, *At1G54620*, *At1G54980*, *At2G15345*, *At3G55680*, *At4G00872*, and *At5G24370* can only be detected in Lineage I, *At1G02550*, *At1G48010*, and *At2G47340* were detected in both Lineage I and *A. arabicum*, and *At1G50325-At1G50340*, *At3G27990*, and *At4G03945* have no orthologous genes in other sequenced Brassicaceae species. These findings indicated that the 12 loci of AtPMEI newly appeared after the separation of *Arabidopsis* and *B. oleracea* (Table S4). In the same manner, we explored the orthologous genes of the 12 BoPMEI loci, whose orthologs were not identified in *A. thaliana*, in other Brassicaceae species. The orthologous genes of eight BoPMEI loci (four were no hits in the searches of BRAD) were only found in *B. rapa* (Table S5), which experienced a WGT event similar to *B. oleracea* [30,31]. These results suggest that the eight loci of BoPMEI were newly acquired after the divergence between *Arabidopsis* and *B. oleracea*, similar to the 12 loci of AtPMEI.

Based on the above results, we computed the retention rate of PMEI genes in *B. oleracea*. The retention rates were analyzed in a set of 458 core eukaryotic genes and 458 randomly selected genes. The results show that a similar number of BoPMEI genes and core eukaryotic genes were retained (50% and 52%). This number was higher than 45% of the randomly selected genes (Figure 3b). Half of BoPMEI genes retained two or three copies, and this number was considerably larger than the 45% result of the randomly selected genes (Figure 3c). This phenomenon was also observed in the core eukaryotic genes. To evaluate the retained proportions of BoPMEI genes in the whole genome level, we calculated these values in the three subgenomes, namely, least fractionated subgenome (LF), medium fractionated subgenome (MF1), and most fractionated subgenome (MF2) (Figure 3d) [21,32]. The BoPMEI gene family retained more genes in the LF subgenome than those in the MF1 or MF2 subgenomes, which was similar to the phenomenon in randomly selected genes and core eukaryotic genes. The BoPMEI genes in the subgenomes of MF1 and MF2 retained similar proportions to core eukaryotic genes (45% and 48%, respectively) and substantially larger than that of randomly selected genes (37%). These findings indicated that the gene retention in the three *B. oleracea* subgenomes was biased. The higher retention rate of BoPMEI genes in the LF subgenome was similar to that of previous reports, which can be explained by a “two-step theory”. This theory states that the MF1 and MF2 subgenomes underwent two rounds of gene fractionation and LF subgenome only underwent one round, so the MF1 and MF2 subgenomes lost more genes than the LF subgenome [24,33].

### 2.6. Evolution Analysis of BoPMEI Gene Family

**Figure 4:**
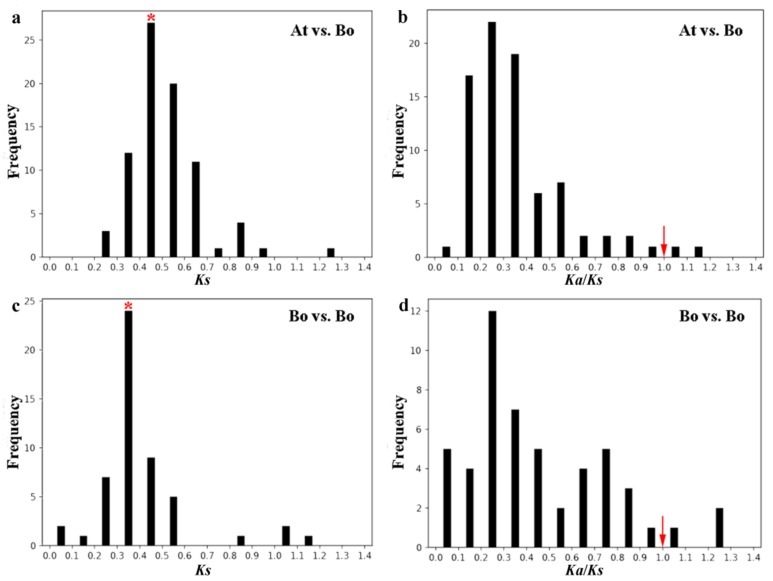
*Ks* and *Ka*/*Ks* distributions of homologous PMEI gene pairs. (**a**,**b**) *Ks* and *Ka*/*Ks* distributions of the PMEI ortholog pairs obtained from the *Brassica oleracea* and *Arabidopsis thaliana* genomes. (**c**,**d**) *Ks* and *Ka*/*Ks* distributions of the BoPMEI paralog pairs. The vertical axes denote the frequency of paired sequences, and the horizontal axes indicate the *Ks* and *Ka*/*Ks* values at 0.1 intervals. The peaks of the bars are marked with a red asterisk which represents the principal distribution extent of the *Ks* values. The red arrows indicate *Ka*/*Ks* = 1. At: *Arabidopsis thaliana*; Bo: *Brassica oleracea*; *Ks*: synonymous substitution rate; *Ka*: nonsynonymous substitution rate.

To investigate the evolution process, we computed the *Ka*, *Ks*, and ω ratio of PMEI ortholog pairs between the *B. oleracea* and *Arabidopsis* genomes as well as PMEI paralog pairs in *B. oleracea.* In all, 81 PMEI ortholog pairs were analyzed (Table S6). As a proxy for time, the relative *Ks* value was used in evaluating the divergence time of homologous gene pairs. Among the 81 PMEI ortholog pairs, the *Ks* values were from 0.2 to 1.3, and most were focused on 0.4–0.5 (Figure 4a) with a duplication time of approximately 13.3–16.7 MYA, which was close to the divergence time between *B. oleracea* and *Arabidopsis* [21]. In addition, the ω ratios of 79 ortholog pairs were lower than 1 (Figure 4b), implying that they underwent purifying selection except for *BoPMEI72* and *BoPMEI75*. Only one pair might be under strong purifying selection stress, with the ω ratio lower than 0.1. Among the three subgenomes, the *Ks* values of ortholog pairs in LF subgenome were higher than that in MF1 subgenome, but no obvious difference existed in the subgenomes of LF and MF2 (Figure S5a), whereas the mean ω ratios of the three subgenomes did not differ significantly (Figure S5b). The *Ks* values of BoPMEI paralog pairs peaked at 0.3–0.4, with an average duplication time of 13.6 MYA (Figure 4c and Table S7), in keeping with the WGT time of *B. oleracea* (13–17 MYA) [21]. Moreover, 48 out of 52 paralog pairs experienced purifying selection, with ω ratios lower than 1 (Figure 4d and Table S7). The average *Ks* value of paralogs was lower than that of orthologs, suggesting shorter divergence time, and the average ω ratio of paralogs was higher than that of orthologs, indicating greater selective constraint.

### 2.7. Expression Profiles of PMEI Genes

**Figure 5:**
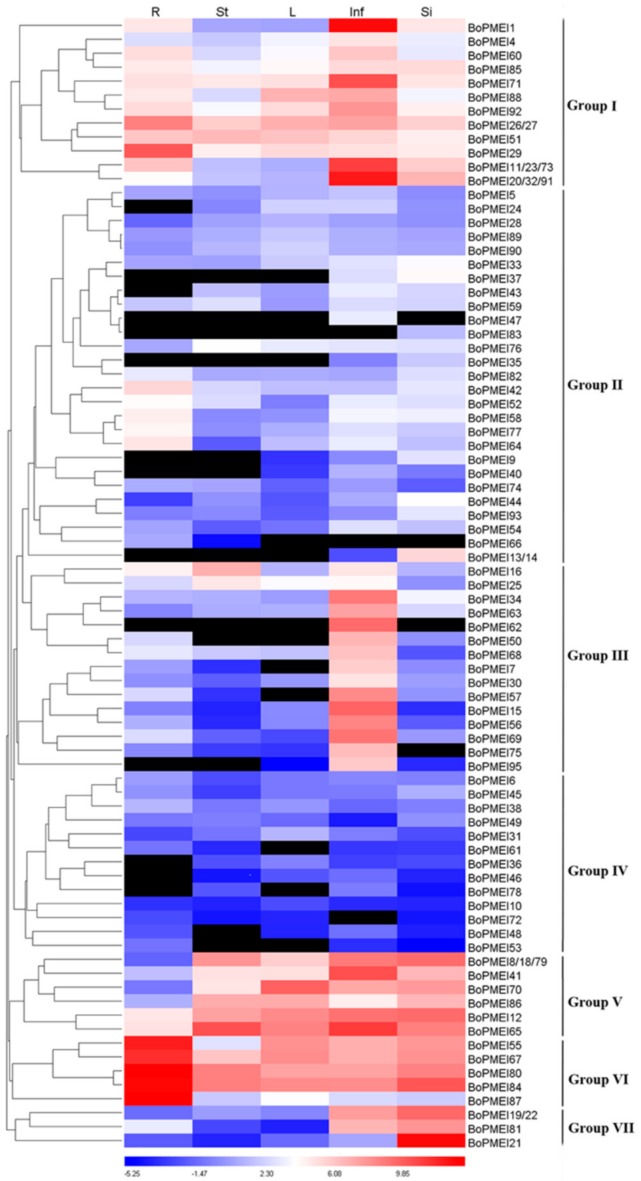
Expression profiles of BoPMEI genes in root (R), stem (St), leaf (L), inflorescence (Inf), and silique (Si) at the flowering stage (approximately 22 weeks after sowing). The gene expression levels in the five tissues were detected by qRT-PCR. The vertical dark bars on the right designate the seven groups of BoPMEI genes. The black box indicates undetectable expression.

Quantitative Real-Time PCR (qRT-PCR) was carried out to estimate the expression levels of 95 BoPMEI genes in five different tissues including root, stem, leaf, inflorescence, and silique at the flowering stage (approximately 22 weeks after sowing). Given that the expression of five BoPMEI genes was not found in any of the five tissues, the expression patterns of 90 genes were analyzed. The 90 BoPMEI genes were classified into seven groups based on the differential expression patterns (Figure 5). Group I contained 17 genes that had specific or high expression levels in inflorescences, although several genes displayed relatively high expression levels in mature roots, such as *BoPMEI26/27*, *BoPMEI51*, and *BoPMEI29*. Group II included 28 genes that showed quite low expression levels in all five tissues, with five genes having relatively high expression levels in mature roots. The 15 genes in Group III were specifically expressed in inflorescences, while *BoPMEI16* and *BoPMEI25* were also largely expressed in mature stems. All genes that belonged to Group IV had low expression levels in the five tissues. Group V included eight genes, which had high expression levels in mature stems, mature leaves, inflorescences, and siliques, whereas the five genes in Group VI displayed the highest expression levels in mature roots. The remaining four BoPMEI genes belonged to Group VII, which showed high expression levels in inflorescences and siliques. In addition, as shown in Figure 5, many duplicated PMEI genes exhibited apparent expression divergence, such as *BoPMEI33*/*BoPMEI67* and *BoPMEI89*/*BoPMEI92*, while some duplicated genes displayed similar expression profiles, such as *BoPMEI21*/*BoPMEI94* and *BoPMEI59*/*BoPMEI82*.

The expression levels of AtPMEI genes in roots, stems, leaves, siliques, and mature pollen were acquired from the Arabidopsis electronic Fluorescent Pictograph (eFP) Browser to explore the differences and similarities in expression profiles between BoPMEI and AtPMEI genes. The expression of 48 out of 79 AtPMEI genes was detected on this website (Figure S6 and Table S8). The expression patterns of approximately half of the BoPMEI genes were different from those of AtPMEI genes, indicating that functional differentiation can occur among these genes during evolution.

### 2.8. Expression Patterns of BoPMEI Genes in Stamen Development

**Figure 6:**
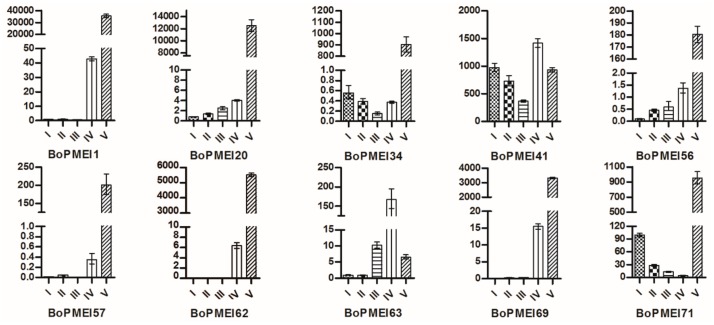
Expression levels of representative BoPMEI genes at five stages (I–V) of flower buds. I, Stage I, pollen mother cell stage; II, Stage II, tetrad stage; III, Stage III, uninucleate microspore stage; IV, Stage IV, binucleate microspore stage; V, Stage V, mature pollen stage.

**Figure 7:**
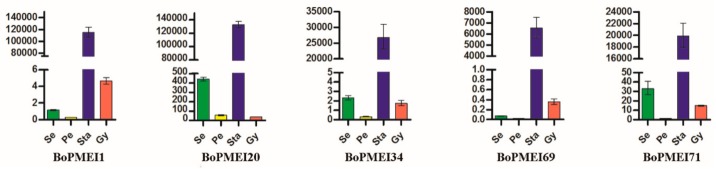
Expression levels of representative BoPMEI genes in sepal (Se), petal (Pe), stamen (Sta), and gynaeceum (Gy).

As shown in Figure 5, many BoPMEI genes had specific or high expression levels in inflorescences. To further elucidate the specific expression patterns of BoPMEI genes in reproductive growth, we selected 10 BoPMEI genes, which were specifically and largely expressed in inflorescences, to detect their expression profiles in subdivided flower buds. The results showed that eight out of 10 BoPMEI genes had relatively high expression levels in flower buds at Stage V, except for *BoPMEI41* and *BoPMEI63*, indicating that these genes might play parts in mature buds (Figure 6). Subsequently, five out of 10 BoPMEI genes, which showed specific and large expression levels in the mature buds, were selected to further analyze their expression patterns in the four floral parts of flower buds at Stage V, containing sepals, petals, stamens, and pistils. As shown in Figure 7, all five BoPMEI genes displayed high relative expression levels in the stamens. Among the 48 AtPMEI genes detected in the Arabidopsis eFP Browser, 12 genes were largely or specifically expressed in mature pollen (Figure S6). Further analysis revealed that the five detected BoPMEI genes, namely, *BoPMEI1*, *BoPMEI20*, *BoPMEI34*, *BoPMEI69*, and *BoPMEI71*, had orthologous relationships with *At4G24640*, *At2G47050*, *At1G10770*, *At3G62180*, and *At1G10770*, respectively (Table S3), which were included in the above-mentioned 12 AtPMEI genes. Thus, we speculated that the five BoPMEI genes might be connected with pollen maturation in the stamen development of *B. oleracea*.

### 2.9. Cis-Elements in the Promoters of BoPMEI Genes

To further investigate the potential functions of BoPMEI genes, the *cis*-elements in promoter sequences of BoPMEI genes were identified and analyzed by PlantCARE. Twenty-five common *cis*-elements related to hormone responses (abscisic acid (ABA), ethylene, gibberellin (GA), indole-3-acetic acid (IAA), Methyl Jasmonate (MeJA), and salicylic acid) as well as the regulation under biotic and abiotic stresses (fungus, high or low temperature, wound, salt, anoxia, light, and drought) were detected (Table S9). Up to 67 promoters of BoPMEI genes contained the GA (GA-respective (GARE)-motif, P-box, and TATC-box) related *cis*-elements. The MeJA (TGACG-motif and CGTCA-motif) and salicylic acid (TCA-element) responsive elements were detected in the promoter regions of 58 and 55 BoPMEI genes, respectively. A total of 47 and 43 promoters of BoPMEI genes included the ABA (ABA-responsive element (ABRE), CE3, and motif IIb) and IAA (AuxRR-core and TGA-element) related *cis*-elements, respectively. Besides, we also identified the ethylene-responsive element (ERE) in 24 promoters of BoPMEI genes. In terms of the responsiveness to various biotic and abiotic stresses, 59 promoters containing the *cis*-acting element related to heat stress responsiveness (HSE) and 40 promoters including the low-temperature responsive element (LTR) were identified. The MYB binding site involved in light responsiveness (MRE) were detected in 32 promoters. The *cis*-element essential for anaerobic induction (anaerobic responsive element, ARE) was detected in up to 78 promoters of BoPMEI genes, whereas only 11 promoters covered the wound-responsive element (wound (WUN)-motif). In addition, 43 promoters possessed Box-W1, a responsive element of fungal induction. In general, the number of the *cis*-elements detected in the promoters of 95 BoPMEI genes was from 1 (*BoPMEI29*) to 31 (*BoPMEI43*). Up to 199 MeJA related *cis*-elements and 169 anaerobic responsive elements were detected in the promoter sequences of 95 BoPMEI genes.

## 3. Discussion

Gene duplications can provide raw materials for adaptive evolution and play significant roles in the rapid expansion of gene families [34]. Generally, the genome duplication event happens more frequently in plants than in other eukaryote species. *Arabidopsis thaliana*, the model plant, has been proven to experience a γ event shared with most dicotyledons and two subsequent genome duplications, namely, α and β events, shared with other Brassicales members [35]. *B. oleracea*, a diploid species, underwent another WGT event besides the three paleo-polyploidy events shared with *Arabidopsis* since its separation from a common ancestor with *Arabidopsis* 13–17 MYA. In this research, 95 PMEI genes were identified in the genome of *B. oleracea* (Table S1), which was more than those identified in *A. thaliana* (79) [19]. The analysis of synteny showed that 77 syntenic PMEI ortholog pairs were identified (Figure 3a and Table S3), which indicated that WGT was the main mechanism accounting for the BoPMEI gene family’s expansion. In addition, by orthologous comparison with closely related Brassicaceae species, we speculated that eight loci of AtPMEI were lost in *B. oleracea*, while 12 AtPMEI loci and eight BoPMEI loci independently appeared after the divergence between *B. oleracea* and *Arabidopsis* (Tables S4 and S5). These findings manifested that the expansion of BoPMEI genes might be due to some other processes except for WGT, suggesting that the birth and death of genes are ongoing. Moreover, the analysis of chromosomal locations showed that 21 out of 95 BoPMEI genes were tandemly duplicated (Figure 1), which indicated that tandem duplication (TD) was another important way for the expansion of PMEI gene family in *B. oleracea*. Thus, from the above-mentioned points, we can conclude that the expansion of BoPMEI genes was mainly attributed to WGT and TD, which was similar to some other gene families, such as the ATP-Binding Cassette (ABC) genes of *B. rapa*, and the pectin methylesterases (PME) genes of cotton [36,37].

Considering that *B. oleracea* experienced an extra WGT event after its separation from *Arabidopsis*, the number of genes in *B. oleracea* should be thrice that of *Arabidopsis* in theory. However, only 45,758 protein-coding genes were identified in the *B. oleracea* genome, which was remarkably fewer than three times of the 27,411 genes in the *A. thaliana* genome [21,24]. In this article, we only found 95 BoPMEI genes (Table S1), which was considerably fewer than the theoretical number. The loss rate of BoPMEI genes was similar to that of genes in the whole *B. oleracea* genome. These results indicated that substantial gene loss can occur in the *B. oleracea* genome after WGT, which is in accordance with the widespread gene fractionation following polyploid formation in other eukaryotes [38]. Moreover, in the aspect of TD, both the number of tandemly duplicated PMEI genes and the mean number of PMEI genes in each tandem cluster in *B. oleracea* were lower than those in *A. thaliana* (Figure 1 and Figure S1), although the total number of BoPMEI genes was more than that of AtPMEI genes. This finding was consistent with that in the whole genome level of *B. oleracea*, in which the triplicated tandem duplication genes were significantly lost since the WGT event, leading to an obviously larger loss proportion of tandem genes than that of non-tandem genes [24]. A previous study also revealed that the WGT event can affect the evolution of tandemly duplicated genes [39]. In addition, as tandem genes underwent a rapid evolution of birth-and-death, they can lose in previous regions and rise in other genomic positions [40]. This feature might result in a decrease of syntenic tandem clusters between *B. oleracea* and *Arabidopsis*. Thus, reasonably, among nine clusters of tandemly duplicated PMEI genes in *A. thaliana*, only four clusters have one or two syntenic PMEI tandem arrays in the three subgenomes of *B. oleracea* (Table S3).

In *B. oleracea*, the PMEI genes were preferentially retained, with a retention rate of up to 50% (Figure 3a). In addition, compared with the randomly selected genes, more (50%) BoPMEI genes retained two or three copies (Figure 3b). The preferential retention of BoPMEI genes can be accounted for the gene balance hypothesis, which predicts that if the products of genes were related to one another or in networks, these genes should be over reserved, for fear of the network imbalances resulted from the disappearance of one member [41,42,43]. In plant cell walls, pectin is one of the most complex polysaccharides, abundant in dicotyledon primary cell walls [44]. Given the structural complexity of pectin, the network of its modification and degradation involves a series of enzymes, such as PMEs, PMEIs, polygalacturonases, and pectate lyases [45]. PMEIs can regulate the activity of PMEs, thereby affecting the release of methanol from methyl-esterified pectin [46]. Thus, PMEIs are closely connected with the metabolic networks of pectins in plant cell walls, essential for plant growth and development.

After the polyploidization following whole-genome duplication, the number of genes can increase simultaneously [47], and then during the long-term evolution, the duplicated genes may experience different fates, such as nonfunctionalization, subfunctionalization, and neofunctionalization [25]. No expression or a relatively weak expression level was detected in many BoPMEI genes in five different tissues (Figure 5), and some genes lost critical motifs (Figure 2), suggesting that nonfunctionalization might occur among these genes. However, the genes showing no or weak expression might also be expressed in other organs or under specific conditions. The ω ratios of four PMEI paralog pairs were more than 1 (Figure 4d and Table S7), indicating that these genes more likely underwent neofunctionalization [48]. Furthermore, many paralogs displayed different spatial expression profiles, and the ω ratios of most paralog pairs were less than 1 (Figure 5d and Figure 6 and Table S7), suggesting that subfunctionalization can be the major driver for the evolution of BoPMEI gene family. Except for the above three fates, the duplicated genes can maintain the same functions, which was called functional conservation [25]. Part of duplicated BoPMEI genes had same expression profiles (Figure 5), indicating their conservative functions. Moreover, the conservative gene structures detected between BoPMEI and AtPMEI genes (Tables S1 and S2) were another line of evidence for functional conservation in BoPMEI genes.

Previous reports showed that PMEI genes played significant roles in the plant vegetative and reproductive growth. Changes in the expression level of specific PMEI genes can affect many growth and developmental processes, including pollen tube elongation [12,14], apical meristems development [49], dark-grown hypocotyl elongation [10], seed maturation [20,50], fruit development [16], and plant-pathogen resistance [11,51,52,53]. As we know, the studies on gene expression profiles can reveal helpful clues to the gene functions. Thus, in this work, we studied the expression patterns of all BoPMEI genes in five different tissues, and most of them (90/95) were expressed in at least one tissue (Figure 5). More than half of BoPMEI genes showed specific or high expression levels in inflorescences and/or siliques, suggesting that these genes might be closely related to the reproductive development of *B. oleracea*. Some BoPMEI genes also showed mature root/stem/leaf-specific expression profiles, implying that they might play a specific part in related tissues. In addition, increasing evidence verified that PMEIs play important roles in responses to various hormones and stresses [11,52,54,55]. Among the promoters of 95 BoPMEI genes, many *cis*-elements involved in the hormone and stress responses were detected (Table S9), indicating that the BoPMEI genes might be closely related to regulating the responses to hormones as well as biotic and abiotic stresses.

In this study, five BoPMEI genes, namely *BoPMEI1*, *BoPMEI20*, *BoPMEI34*, *BoPMEI69*, and *BoPMEI71*, showed high relative expression levels in the stamens of flower buds in Stage V (Figure 7). In addition, 12 AtPMEI genes were largely or specifically expressed in mature pollen (Figure S6). Based on the orthologous relationships between the five detected BoPMEI genes and four out of 12 AtPMEI genes (Table S3), we speculated that these five BoPMEI genes might play roles in pollen maturation. In addition, the genes specifically expressed in the late pollen development can participate in the regulation of pollen tube growth [12,56]. *BoPMEI71*, namely, *Bol031283*, has essential roles in pollen tube growth [12]. Transferring the *Bol031283* antisense gene to *Arabidopsis* plants can suppress the expression level of its ortholog, namely, *At1g10770*, ti retard pollen tube growth, and result in partial male sterility. Among the 12 AtPMEI genes, *At3G17220*, namely, *AtPMEI2*, is closely related to the pollen tube growth [57]. These findings suggested that the five BoPMEI genes might play roles in stamen development, especially in mediating the pollen maturation and/or pollen tube growth of *B. oleracea*. However, more experiments should be conducted to determine the specific functions of these BoPMEI genes.

## 4. Materials and Methods

### 4.1. Plant Materials

Cabbage “Sanxiong” (*Brassica oleracea* L. var. *capitata* L. cv. Sanxiong) plants were grown on an experimental farm in Zhejiang Academy of Agricultural Sciences, China. At the flowering stage (approximately 22 weeks after sowing), the roots, stems, leaves, inflorescences, and siliques were sampled to analyze the tissue-specific expression levels [58]. According to the results of cytological examination in a previous study [59], we divided the flower buds into five stages: Stage I, pollen mother cell stage; Stage II, tetrad stage; Stage III, uninucleate microspore stage; Stage IV, binucleate microspore stage; and Stage V, mature pollen stage. The flower buds corresponding to the five different stages of pollen developmental and the four floral parts of the flower buds at Stage V, including sepals, petals, stamens, and pistils, were collected. The materials were frozen in liquid nitrogen and then stored at −80 °C until further processing.

### 4.2. Identification of PMEI Family Members in B. oleracea

Protein sequences of 79 PMEI genes in *Arabidopsis* [19] downloaded from the The Arabidopsis Information Resource (TAIR) website (http://www.arabidopsis.org/) were used as queries in performing BLASTP (Basic Local Alignment Search Tool of Protein) searches with default algorithm parameters on the *Brassica* database (BRAD, http://brassicadb.org/brad/). The protein sequences of *Brassica* genome were downloaded from BRAD, the HMM pattern of PMEI domain (Pfam04043) was downloaded from the Pfam (http://pfam.xfam.org) [60] database, and the software of HMMER 3.0 was used to search putative PMEI genes with a default value. Some candidate members were identified after removing the redundant sequences. Finally, to validate whether these sequences possess the conserved PMEI domain, we checked the identified sequences using Pfam and Simple Modular Architecture Research Tool (SMART, http://smart.embl-heidelberg.de/) [61] databases. The PMEI genes identified in the genome of *B. oleracea* were named according to their locations and orders on the chromosomes or scaffolds.

### 4.3. Chromosomal Localization and Phylogenetic Analysis

The locations of PMEI genes on the chromosomes or scaffolds in *B. oleracea* were obtained from the *Brassica* Genome Browse (http://brassicadb.org/cgi-bin/gbrowse/Brassica_v1.5/), and the map was drawn using the software of Mapinspect and Adobe Photoshop CC. The Chromosome Map Tool (http://www.arabidopsis.org/jsp/ChromosomeMap/tool.jsp) in TAIR was used to draw the map of PMEI genes in *Arabidopsis*.

The MEGA 6.0 software was used to conduct phylogenetic analysis by the neighbor-joining algorithm with 1000 bootstraps, the “Pairwise deletion” option, and “Poisson model”, based on the protein sequences [62].

### 4.4. Analysis of Gene Structure, Motif Recognition, and Physicochemical Property

The genomic, coding, and protein sequences of BoPMEI genes were downloaded from BRAD. Gene Structure Display Server 2.0 (GSDS 2.0, http://gsds.cbi.pku.edu.cn/index.php) [63] was used to illustrate the organizations of exons and introns for PMEI genes in *B. oleracea* and *Arabidopsis*. The intron phases were also investigated based on exon information. Phase 0 is assigned to introns located between codons, phase 1 is assigned to introns located between the first and second nucleotides of a codon, and phase 2 is assigned to introns located between the second and third nucleotides. Multiple Em for Motif Elicitation (MEME Version 4.11.4, http://meme-suite.org/tools/meme) [64] was used to identify the conserved motifs of the BoPMEI protein sequences with default parameters except for the following aspects: number of repetitions, any; maximum number of motifs, 10; minimum motif width, 6; and maximum motif width, 50 [26]. ProtParam (http://web.expasy.org/protparam/) was used to compute theoretical isoelectric points and molecular weights, and SignalP 4.1 Server (http://www.cbs.dtu.dk/services/SignalP/) was used to explore the signal peptide sequences. The transmembrane helices in PMEI proteins were predicted by trans-membrane hidden Markov model (TMHMM) Server V2.0 (http://www.cbs.dtu.dk/services/TMHMM/) [65]. Plant-mPLoc (http://www.csbio.sjtu.edu.cn/bioinf/plant-multi/), WoLF PSORT (https://wolfpsort.hgc.jp/) and ProtComp 9.0 (http://linux1.softberry.com/berry.phtml?topic =protcomppl&group=programs&subgroup=proloc) were used to predict the subcellular localization of PMEI proteins [66]. The solubility of proteins overexpressed in the bacterium *Escherichia coli* was predicted by the website of http://www.biotech.ou.edu/ [67].

### 4.5. Synteny and Retained Rate Analysis

The syntenic relationships of the BoPMEI and AtPMEI genes were confirmed by searching “syntenic gene” (http://brassicadb.org/brad/searchSyntenytPCK.php) in BRAD [27]. The chromosomal locations of BoPMEI and AtPMEI genes were collected from TAIR and BRAD, respectively. Finally, the colinearity of PMEI genes in or between both genomes was revealed by Circos 5.05 [68].

For the analysis of retention rate, the term “locus” was used instead of “gene” according to a previous research to eliminate the disturbance of TD after the WGT event [69]. A set of 458 core eukaryotic genes and 458 randomly selected genes in *Arabidopsis* were downloaded from Core Eukaryotic Genes Mapping Approach (CEGMA, http://korflab.ucdavis.edu/Datasets/cegma/) and were used for the retained proportion analysis [70]. The orthologous genes of some AtPMEI and BoPMEI genes in *Aethionema arabicum*, *Arabidopsis lyrata*, *Brassica napus*, *Brassica rapa*, *Capsella rubella*, *Camelina sativa*, *Leavenworthia alabamica*, *Thellungiella halophila*, *Thellungiella salsuginea*, *Sisymbrium irio*, and *Schrenkiella parvula* were also identified by syntenic analysis in BRAD.

### 4.6. Evolutionary Analysis

The *Ka*, *Ks*, and ω ratio were calculated to investigate the molecular evolution of homologous gene pairs. Full-length protein sequence alignment was performed by Clustal Omega (http://www.ebi.ac.uk/Tools/msa/clustalo/), and the values of *Ka, Ks*, and ω ratio were computed using PAL2NAL by the codeml program in Phylogenetic Analysis by Maximum Likelihood (PAML) program (http://www.bork.embl.de/pal2nal/index.cgi?example=Yes#RunP2N) [71,72]. The divergence time of homologous gene pairs was calculated by the following equation: T = *Ks*/2R (R = 1.5 × 10^−8^ for dicotyledonous) [73].

### 4.7. RNA Isolation and qRT-PCR

The total RNA was isolated from the materials aforementioned using Trizol reagent (Invitrogen, Carlsbad, CA, USA) treated with DNAase following the manufacturer-recommended protocol [74]. Agarose gel was used to test the RNA integrity. The first strand of complementary DNA was synthesized using a PrimerScript Real-Time (RT) reagent kit (Takara, Shiga, Japan) according to the manufacturer’s instructions. qRT-PCR was conducted to measure the expression levels of BoPMEI genes by using the SYBR^®^ Premix Ex Taq Kit (TOYOBO, Osaka, Japan) with CFX96 Real-Time System (Bio-Rad, Hercules, CA, USA). The qRT-PCR was performed in triplicate by the gene-specific primers (Table S10), which were devised by the Primer Premier 5.0 software. Each primer was examined in BRAD to verify its specificity. *B. oleracea glyceraldehyde*-*3*-*phosphate dehydrogenase* (*BoGAPDH)* was used as the internal control [75]. The relative expression levels of BoPMEI genes were computed by the 2^−ΔΔCt^ method [76], and the results of qRT-PCR were displayed in a heat map generated with Heatmap Illustrator (HemI 1.0, http://hemi.biocuckoo.org/index.php) (log_2_-transformed) [77].

The microarray data for AtPMEI genes were collected from the Arabidopsis eFP Browser (http://bar.utoronto.ca/efp_arabidopsis/cgi-bin/efpWeb.cgi) [78]. Given that the database did not include the data that were completely in conformity with the organs and stages for the expression of BoPMEI genes, the data for root before bolting, stem at the second internode, cauline leaf, and silique at seed Stage 3 were collected. We also gathered the data of mature pollen in *A. thaliana*. These data were calculated similarly as that for BoPMEI genes.

### 4.8. Cis-Elements Analysis

To investigate the *cis*-elements in promoter regions of BoPMEI genes, the upstream sequences (1.5 kb) of the initiation codon (ATG) for each gene were obtained from BRAD. The PlantCARE (Cis-Acting Regulatory Element) database (http://bioinformatics.psb.ugent.be/webtools/plantcare/html/) [79] was used to analyze the *cis*-elements in promoter regions.

## 5. Conclusions

In this paper, we identified 95 PMEI genes in the genome of *B. oleracea*. The phylogenetic tree, protein domains, intron-exon structures, physicochemical properties, and promoters, were investigated systematically. In addition, the analyses of collinearity of homologous genes and chromosome distributions suggested that WGT and TD were the main mechanisms accounting for the expansion of BoPMEI gene family. The ω ratios of most homologous gene pairs were lower than 1, suggesting that they evolved through purifying selection after the duplication events. Preferential and biased retention was found in the evolutionary process of BoPMEI genes, which can be explained by the gene balance hypothesis. We also identified five BoPMEI genes that might specifically contribute to the regulation of pollen maturation and/or pollen tube growth. Our findings will provide a better understanding of the molecular evolution of the PMEI genes in *B. oleracea* and offer a good foundation for the further research of the PMEI genes in plants.

## Supplementary Materials

Supplementary materials can be found at http://www.mdpi.com/1422-0067/19/11/3338/s1.

## Abbreviations

BRAD	Brassica database
HMM	hidden Markov model
*Ka*	nonsynonymous substitution rate
*Ks*	synonymous substitution rate
LF	least fractionated subgenome
MEME	multiple Em for Motif Elicitation
MF1	medium fractionated subgenome
MF2	most fractionated subgenome
MYA	million years ago
PMEIs	pectin methylesterase inhibitors
PMEs	pectin methylesterases
qRT-PCR	quantitative real-time PCR
TAIR	The Arabidopsis Information Resource
TD	tandem duplication
WGT	whole genome triplication
